# Revealing the Toxicity-Enhancing Essence of Glycyrrhiza on *Genkwa Flos* Based on Ultra-high-performance Liquid Chromatography Coupled With Quadrupole-Orbitrap High-Resolution Mass Spectrometry and Self-Assembled Supramolecular Technology

**DOI:** 10.3389/fchem.2021.740952

**Published:** 2021-12-23

**Authors:** Yuqin Yang, Feifei Li, Mengmeng Yan, Shan Chen, Desheng Cai, Xiaojing Liu, Nana Han, Zhihua Yuan, Jihui Lu, Yaozhi Zhang, Qiang Ma, Penglong Wang, Haimin Lei

**Affiliations:** ^1^ School of Chinese Materia Medica, Beijing University of Chinese Medicine, Beijing, China; ^2^ Chinese Academy of Inspection and Quarantine, Beijing, China

**Keywords:** genkwa flos, glycyrrhizic acid, ultra-high-performance liquid chromatography coupled with quadrupole-orbitrap high-resolution mass spectrometry, self-assembled supramolecular technology, toxicity

## Abstract

Researchers often focus on the mechanisms of synergistic agents, a few explore drug combinations that enhance toxicity, while few have studied the internal mechanism of compatibility enhancement in chemical level. Herein, we present a comprehensive analysis based on ultra-high-performance liquid chromatography coupled with quadrupole-Orbitrap high-resolution mass spectrometry (UHPLC-Q-Orbitrap HRMS) and a self-assembled supramolecular strategy, which reveals the toxicity-enhancing essence of glycyrrhizic acid originated in licorice when combined with *Genkwa Flos*. Through this method, we discovered the toxicity was enhanced through the formation of a supramolecular complex from *Genkwa Flos*/glycyrrhizic acid. The morphology and size distribution of the self-assembled nanoparticles were characterized by scanning electron microscopy and dynamic light scattering Furthermore, a total of 58 constituents (eight diterpenoids, 35 flavonoids, five phenylpropanoids, four nucleosides, two amino acids, and four other compounds) consisted from the supramolecular complex were identified through accurate-mass measurements in full-scan MS/data-dependent MS/MS mode. Based on the hydrophobic interaction of glycyrrhizic acid with yuanhuacine (one of main ingredients from *Genkwa Flos*), the supramolecular self-assembly mechanism was revealed with proton nuclear magnetic resonance (^1^H-NMR) and NOESY 2D NMR. The toxicity of *Genkwa Flos* and *Genkwa Flos*/glycyrrhizic acid supramolecular complex were compared through *in vitro* studies on L-02 cells using a 3-(4,5-dimethylthiazol-2-yl)-2,5-diphenyltetrazolium bromide (MTT) assay; and 4′,6-diamidino-2-phenylindole (DAPI) staining was performed to further confirm the enhancement inhibition of *Genkwa Flos*/glycyrrhizic acid supramolecular complex than *Genkwa Flos*. This study provides fundamental scientific evidence of the formation of a self-assembled phytochemical supramolecular when *Genkwa Flos* and glycyrrhizic acid are combined, enabling to understand their clinical incompatibility and contraindication.

## Introduction

Herbs comprise the vast majority of plant species and are used collectively to enhance therapeutic efficacy and reduce adverse effects based on clinical experience. In traditional medicine, licorice can either harmonize and modify herbs in a prescription (Zhu et al., 2017) (Zhu et al., 2017)^,^ (Guo et al., 2014)], or it can enhance the toxicity of herbs such as *Herba CirsiiJaponici*, *Euphorbia kansui* (Chen et al., 2019a) and *Genkwa Flos*. When licorice is used jointly with *Genkwa Flos*, the incompatible licorice–*Genkwa Flos* herbal pair is believed to exacerbate toxic effects^[^2^]^, enhancing the inhibitory action of P-glycoproteins (Peng et al., 2018), which is clinically acceptable (Yu et al., 2017; Chen et al., 2019b; Yu et al., 2019). Previous studies have inferred that glycyrrhizic acid (the main active compounds in licorice) and terpene alcohol ester may form a micelle to increase the dissolution of the toxic component (Polyakov et al., 2008; Selyutina et al., 2016; Shen et al., 2020; You et al., 2021) (Polyakov et al., 2008; Selyutina et al., 2016; Shen et al., 2020; You et al., 2021)]. However, glycyrrhizic acid has not been directly observed to increase the dissolution of terpene alcohol ester in that form. Moreover, research on supramolecular phytochemicals that increase toxicity is also unclear.

Supramolecular self-assembly based on host-guest interactions is a process of spontaneous formation of unique nanostructures by dynamic covalent interactions (Wang et al., 2018) (Wang et al., 2018)and non-covalent intermolecular interactions (Caulder and Raymond, 1999; Okesola et al., 2019; Guo et al., 2018). Recently, supramolecular assemblies in traditional medicines have received more attention because they can improve treatment efficacy and reduce toxicity. Inspired by natural supramolecular assembly, our previous studies have found a variety of self-assembled supramolecular structures, including hydrogels (Li et al., 2019) (Li et al., 2019), nanoparticles/microparticles (Tian et al., 2020), and crystals (Huang et al., 2019), formed through various combinations of traditional medicines. Most recently, we found that glycyrrhizic acid can self-assemble with compounds in *Genkwa Flos* to form a kind of spherical supramolecular structure (*Genkwa Flos*/glycrrrhizic acid self-assembled complex), which had not been detected in previous studies of their mechanism or chemical composition.

Herbs have complex chemical systems involving various hydrophobic, hydrophilic, and amphiphilic molecules that feature a wide variety of acid-base properties, polarity, molecular mass, and content [(Luo et al., 2020), (Wang et al., 2020)], making it difficult to analyze their composition [(Feng et al., 2021), (Ji et al., 2018)]. In most previous studies, the systematic and comprehensive analysis of herb composition through liquid chromatography mass spectrometry had limited success due to limitations such as low precision and high detection limits [(Liu et al., 2020a)]. Fortunately, the ultra-high-performance liquid chromatography coupled with quadrupole-Orbitrap high-resolution mass spectrometry (UHPLC-Q-Orbitrap HRMS) platform is a powerful tool for the comprehensive identification and analysis of ingredients in herbs due to its higher mass resolution, higher mass accuracy, wider dynamic range, and better sensitivity (Liu et al., 2020b; Xian et al., 2020; Yuan et al., 2020; Jia et al., 2021).

In this study, we utilized UHPLC-Q-Orbitrap HRMS to analyze the composition of a self-assembled supramolecular structure. In combination with proton nuclear magnetic resonance (^1^H-NMR), NOESY 2D NMR, scanning electron microscopy (SEM), and cytotoxicity test, we discuss the self-assembly mechanism and reveal the toxicity-enhancing essence of glycyrrhizic acid on *Genkwa Flos*. This study provides fundamental scientific evidence of the presence of a *Genkwa Flos*/glycyrrhizic acid self-assembled phytochemical supramolecular, enabling better understanding of their clinical incompatibility and contraindication.

## Experimental

### Reagents and Materials

*Genkwa Flos* was purchased from Beijing Tong Ren Tang (Beijing, China). Glycyrrhizic acid was supplied by Shanghai Yuanye Bio-Technology Co., Ltd. (Shanghai, China), with the purity greater than 98%. MS-grade methanol was purchased from Merck (Darmstadt, Germany). Ultrapure water was prepared using a Milli-Q Integral 5 water purification system (Millipore, Bedford, MA, United States ).

### Sample Preparation

*Genkwa Flos* (50 g) was packed in a nonwoven bag (5 cm × 7 cm, pore diameter <30 μm) and transferred into a 1000-ml round-bottom flask. Water (500 ml) was added and then heated for 1 h under reflux. The filtrate of the *Genkwa Flos* solution was concentrated using a Rotavapor R-215 rotary evaporator (BUCHI, Flawil, Switzerland) to a volume of 50 ml. The solution was then transferred to a separatory funnel and extracted with 50 ml of ethyl acetate three times. The ethyl acetate extract was lyophilized using a Martin Christ Beta 2–8 LD plus freeze dryer (Osterode, Germany). The resulting *Genkwa Flos* extract powder (25 mg) was combined with 20 mg of glycyrrhizic acid in a 100-ml round-bottom flask. Water (10 ml) was added, and then extracted with reflux for 1 h to obtain a sample of *Genkwa Flos*/glycyrrhizic acid self-assembled complex. In addition, 25 mg of the ethyl acetate extracted *Genkwa Flos* powders was extracted separately as a parallel control for morphology characterization, UHPLC-Q-Orbitrap HRMS analysis and cell experiments. All samples prepared were stored in the dark at 4 °C.

### Characterization of *Genkwa Flos* and the *Genkwa Flos*/Glycyrrhizic Acid Self-Assembled Complex

The morphology and size distribution of the *Genkwa Flos* and the *Genkwa Flos*/glycyrrhizic acid self-assembled complex were characterized by scanning electron microscopy (SEM) and dynamic light scattering (DLS). The yuanhuacine/glycyrrhizic acid self-assembled complex was analyzed by ^1^H-NMR and NOESY 2D NMR.

### Ultra-High-Performance Liquid Chromatography Coupled Quadrupole-Orbitrap High-Resolution Mass Spectrometry Analysis

UHPLC separation was performed on a Thermo Scientific UltiMate 3,000 liquid chromatographic system (Sunnyvale, CA, United States). Chromatographic separation was conducted on a Waters ACQUITY UPLC BEH C_18_ column (150 mm × 2.1 mm, 1.7 μm) at a flow rate of 0.3 ml/min and maintained at 35°C. The mobile phase was composed of a water solution (A) and methanol (B). The elution conditions were set as follows: 0–3 min, 10–70% B; 3–10 min, 70–90% B; 10–15 min, 90% B. A sampling volume of 2 μl was injected for each run. The chromatograph was coupled with a Thermo Scientific Q Exactive quadrupole-Orbitrap mass spectrometer (Bremen, Germany). Positive electrospray ionization (ESI^+^) mode was used, with a spray voltage of 3.5 kV. The capillary gas heater temperature was set to 320°C. The flow rates of sheath gas, auxiliary gas, and sweep gas were 40, 10, and 1 (in arbitrary units), respectively. Full-scan data within the range of *m/z* (mass-to-charge ratio) 50–750 were acquired at a mass resolution of 70,000 FWHM defined for *m/z* 200 at a scan rate of 3 Hz. Data-dependent MS/MS (dd-MS^2^) scan mode was set at a mass resolution of 17,500 FWHM. The stepped normalized collision energies (NCEs) were 20, 40, and 60 eV. The automatic gain control (AGC) target value was set at 1 × 10^5^, with a maximum injection time of 50 ms. Data acquisition and processing were carried out using Xcalibur version 2.3 and TraceFinder version 4.1 software (Thermo Scientific, Bremen, Germany).

### Cell Culture

L-02 (human normal hepatocyte) was obtained from the Chinese Academy of Medical Sciences and Peking Union Medical College. The cell lines were maintained in RPMI-1640 supplemented with 10% (v/v) fetal bovine serum (FBS) and 1% (v/v) penicillin/streptomycin (Corning, New York, NY, United States ) under a humidified atmosphere containing 5% CO_2_ at 37°C. The *Genkwa Flos* and *Genkwa Flos*/glycyrrhizic acid self-assembled complex were dissolved in deionized water and added at required concentrations to the cell culture. Cells incubated without any additions served as the control.

### Cytotoxicity Assay

The cytotoxicities of *Genkwa Flos* and *Genkwa Flos*/glycyrrhizic acid self-assembled complex were evaluated on L-02 cell lines *in vitro* using the MTT method. In short, cells growing in the logarithmic phase were seeded into 96-well plates at a density of 3 × 10^3^ cells/well and incubated for 24 h. Then, each well was exposed to different concentrations of the tested samples of *Genkwa Flos and Genkwa Flos*/glycyrrhizic acid self-assembled complex (100 μl) and incubated for 48 and 72 h at 37°C with 5% CO_2_, respectively. Later, MTT solution (20 μL, 5 mg/ml) was added to each well and incubated for another 4 h. After removing the supernatant medium, 150 μl of dimethyl sulfoxide (DMSO) was added. The optical density (OD) values of the studied samples were measured at 490 nm with a plate reader (BIORAD 550 spectrophotometer, Biorad Life Science Development Ltd., Beijing, China). Wells without drugs were used as blanks. The cell viability (%) was calculated with the following equation:

% cell viability = (Sample group OD - Blank group OD)/(Control group OD - Blank group OD) × 100% (Zhu et al., 2017)

### DAPI Staining

L-02 cells in the logarithmic growth phase were seeded in 12-well plates (2.4 × 10^4^ cells/well). After incubation for 24 h at 37°C with 5% CO_2_, 0.15625 mg/ml of *Genkwa Flos* and *Genkwa Flos*/glycyrrhizic acid self-assembled complex were added to each well, and the plate was incubated for another 72 h. After discarding the cell culture medium, they were washed three times with cold PBS, fixed with 4% paraformaldehyde for 10 min, washing three times with cold PBS again, then stained with 4’,6-diamidino-2-phenylindole (DAPI, 10 μg/ml, Solarbio Life Science, Beijing, China) for 3 min in the dark. Cell nuclei were observed under a fluorescence microscope.

### Statistical Analysis

All data were expressed as mean ± standard deviation (SD) of three replicates. Statistical analysis was performed by SPSS software (version 20.0) to analyze variance. Independent samples *t*-test was used to compare the means between two independent samples. A *p*-value of less than 0.05 was considered significant.

## Results and Discussion

### Size Distribution and Morphology Characterization of *Genkwa Flos* and *Genkwa Flos*/Glycyrrhizic Acid Self-Assembled Complex

**FIGURE 1:**
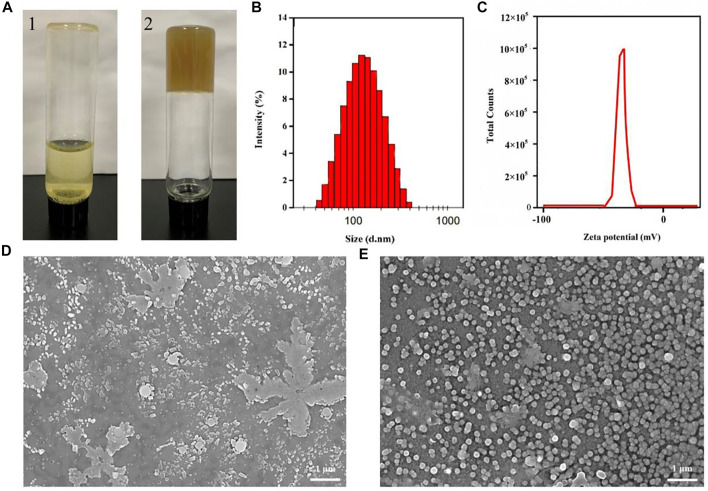
Microscopic morphology of the self-assembled complex. **(A)** Photographs of self-assemblies (1: *Genkwa Flos*; 2: the hydrogel of *Genkwa Flos* and glycyrrhizic acid). **(B)** Particle size of the *Genkwa Flos*/glycyrrhizic acid self-assembled complex. **(C)** Zeta potential of the *Genkwa Flos*/glycyrrhizic acid self-assembled complex. SEM images of **(D)** *Genkwa Flos* and **(E)** *Genkwa Flos*/glycyrrhizic acid self-assembled complex.

**FIGURE 2:**
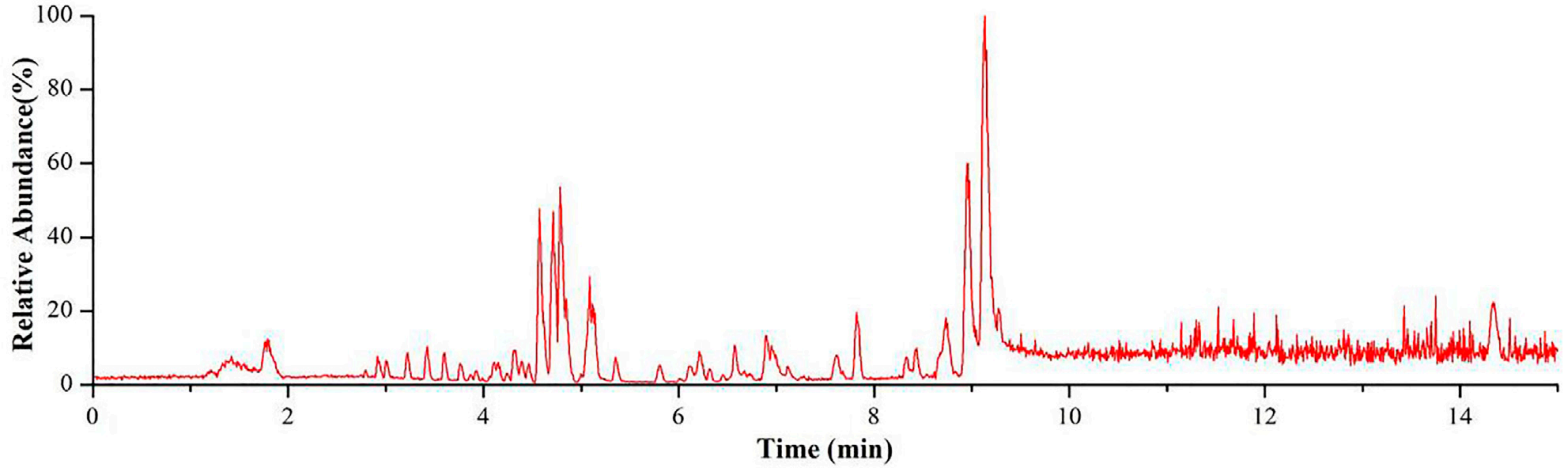
TICC of the *Genkwa Flos*/glycyrrhizic acid self-assembled complex obtained in ESI^+^ mode.

During the decoction process of *Genkwa Flos* and glycyrrhizic acid, we discovered that *Genkwa Flos* and glycyrrhizic acid could self-assemble into a hydrogel in an aqueous solution, which was confirmed by the inverted test tube method (Figure 1A, Figure 2). The size distribution of the *Genkwa Flos*/glycyrrhizic acid nanoparticles was determined with DLS, demonstrating an average particle size of 114 nm (Figure 1B). The zeta potential (ZP) of the *Genkwa Flos*/glycyrrhizic acid self-assembled complex was compared with that of *Genkwa Flos* results, which were −44.6 ± 2.9 mV (Figure 1C) and −34.5 ± 0.8 mV (Supplementary Figure S1), respectively. A ZP value of around ±40 mV typically represents a stabilized self-assembly. In addition, the morphology of *Genkwa Flos* and *Genkwa Flos*/glycyrrhizic acid self-assembled complex was characterized by SEM. The *Genkwa Flos*/glycyrrhizic acid self-assembled complex appeared to be uniform spherical nanoparticles (Figure 1E). The SEM image of *Genkwa Flos* is shown in Figure 1D, which did not display any self-assembly properties. Therefore, after *Genkwa Flos* was decocted with glycyrrhizic acid, stable homogeneous nanoparticles were formed.

### Identification of the Constituents in the *Genkwa Flos*/Glycyrrhizic Acid Self-Assembled Complex

In this study, a total of 58 constituents were tentatively identified in the *Genkwa Flos*/glycyrrhizic acid self-assembled complex, including eight diterpenoids, 35 flavonoids, five phenylpropanoids, four nucleosides, two amino acids, and four other compounds (*e.g.,* alkaloids and terpenoids). The MassBank of North America (MoNA) was also used to identify the compounds in the *Genkwa Flos*/glycyrrhizic acid self-assembled complex. The total ion current chromatogram (TICC) of the *Genkwa Flos*/glycyrrhizic acid self-assembled complex in the ESI^+^ mode is shown in Figure 2. The chemical structures of all constituents are shown in Supplementary Figure S2–S7.

#### Diterpenoids

**FIGURE 3:**
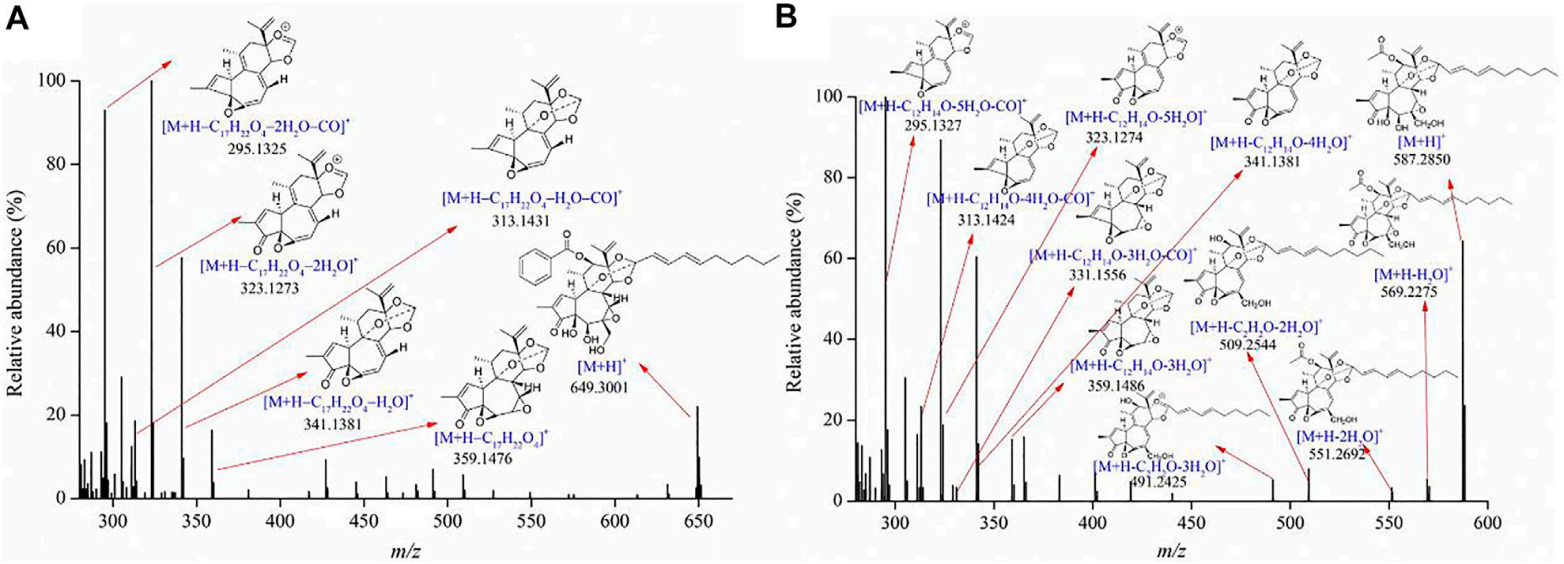
Product ion spectra of **(A)** yuanhuacine and **(B)** yuanhuadine.

Diterpenoids were the major bioactive compounds identified in the *Genkwa Flos*/glycyrrhizic acid self-assembled complex, including yuanhuacine, yuanhuadine, yuanhuafine, yuanhuatine, yuanhuapine, genkwadaphnin, daphne diterpene ester-7, and genkwanine H (Table 1). Diterpenoids have displayed some common mass spectrometric fragmentation features, such as neutral losses of CO and H_2_O. For example, compounds 55 and 54 were characterized to be yuanhuacine (C_37_H_44_O_10_) and yuanhuadine (C_32_H_42_O_10_), respectively. A protonated molecule of yuanhuacine ([M+H]^+^) with a *m/z* of 649.3001 was detected in the positive ion mode. The protonated molecule lost a C_17_H_22_O_4_ moiety, forming a fragment ion [M+H–C_17_H_22_O_4_]^+^ at *m/z* 359.1476. By further losses of H_2_O and CO, product ions [M+H–C_17_H_22_O_4_–H_2_O]^+^ at *m/z* 341.1381 [M+H–C_17_H_22_O_4_–H_2_O–CO]^+^ at *m/z* 313.1431, [M+H–C_17_H_22_O_4_–2H_2_O]^+^ at *m/z* 323.1273 and [M+H–C_17_H_22_O_4_–2H_2_O–CO]^+^ at *m/z* 295.1325 were observed. As for yuanhuadine, a protonated molecule [M+H]^+^ at *m/z* 587.2848 was detected in the positive ion mode. By eliminating H_2_O moieties, fragment ions [M+H–H_2_O]^+^ at *m/z* 569.2275 and [M+H–2H_2_O]^+^ at *m/z* 551.2692 were generated. Yuanhuadine could also produce fragment ions at *m/z* 509.2544 [M+H–C_2_H_2_O–2H_2_O]^+^ and at *m/z* 491.2425 [M+H–C_2_H_2_O–H_2_O]^+^ by losing C_2_H_2_O and H_2_O. The protonated molecule lost C_12_H_14_O, H_2_O, and CO moieties, resulting in fragment ions at *m/z* 359.1486 ([M+H–C_12_H_14_O–3H_2_O]^+^), *m/z* 341.1381 ([M+H–C_12_H_14_O–4H_2_O]^+^), *m/z* 331.1556 ([M+H–C_12_H_14_O–3H_2_O–CO]^+^), *m/z* 323.1274 ([M+H–C_12_H_14_O–5H_2_O]^+^), *m/z* 313.1424 ([M+H–C_12_H_14_O–4H_2_O–CO]^+^), and *m/z* 295.1327 ([M+H–C_12_H_14_O–5H_2_O–CO]^+^). The possible fragments for yuanhuacine and yuanhuadine are proposed in Figure 3. The proposed fragmentation patterns of yuanhuafine, yuanhuatine, yuanhuapine, genkwadaphnin, daphne diterpene ester-7, and genkwanine H are shown in Supplementary Figure S2.

**TABLE 1:** Information on the constituents identified in *Genkwa Flos*/glycyrrhizic acid self-assembled complex in positive ionization mode.

No	Compounds	Formula	Retention time (min)	Identity	Precursor ion			Fragment ions (*m/z*)
					Experimental (*m/z*)	Theoretical (*m/z*)	Mass accuracy (△ppm)	
1	trigonelline	C_7_H_7_NO_2_	0.86	[M+H]^+^	138.0549	138.0550	–0.36	94.0653, 79.0417
2	adenine	C_5_H_5_N_5_	0.93	[M+H]^+^	136.0617	136.0618	–0.73	119.0353
3	phenylacetaldehyde	C_8_H_8_O	1.36	[M+H]^+^	121.0648	121.0648	0.00	103.0543, 93.0701
4	L-leucine	C_6_H_13_NO_2_	1.45	[M+H]^+^	132.1018	132.1019	–0.76	86.0965, 69.0700
5	L-phenylalanine	C_9_H_11_NO_2_	1.70	[M+H]^+^	166.0862	166.0863	–0.60	120.0808, 103.0543
6	guanosine	C_10_H_13_N_5_O_5_	1.84	[M+H]^+^	284.0987	284.0989	–0.70	152.0568, 135.0302, 110.0350
7	iso-guanosine	C_10_H_13_N_5_O_5_	1.84	[M+H]^+^	284.0987	284.0989	–0.70	152.0568, 110.0350
8	adenosine	C_10_H_13_N_5_O_4_	1.86	[M+H]^+^	268.1036	268.1040	–1.49	268.1046, 136.0619
9	chlorogenic acids	C_16_H_18_O_9_	2.26	[M+H]^+^	355.1020	355.1024	–1.13	163.0389, 145.0285, 135.0441, 117.0334, 79.0544
10	neochlorogenic acid	C_16_H_18_O_9_	2.26	[M+H]^+^	355.1020	355.1024	–1.13	163.0389, 145.0285, 135.0441, 117.0334, 79.0544
11	cryptochlorogenic acid	C_16_H_18_O_9_	2.26	[M+H]^+^	355.1020	355.1024	–1.13	163.0389, 145.0285, 135.0441, 117.0334, 79.0544
12	scopolin	C_16_H_18_O_9_	2.26	[M+H]^+^	355.1020	355.1024	–1.13	163.0389, 135.0441, 145.0285, 117.0334, 89.0385
13	7-hydroxycoumarin	C_9_H_6_O_3_	2.28	[M+H]^+^	163.0387	163.0390	–1.84	145.0283, 135.0442, 53.0390
14	schaftoside	C_26_H_28_O_14_	3.51	[M+H]^+^	565.1544	565.1551	–1.24	403.1004, 313.0697, 271.0598
15	diosmetin	C_16_H_12_O_6_	3.80	[M+H]^+^	301.0700	301.0707	–2.33	286.0469, 258.0520, 241.0480, 135.0440, 124.0156
No	Compounds	Formula	Retention time (min)	Identity	Precursor ion			Fragment ions (*m/z*)
					Experimental (*m/z*)	Theoretical (*m/z*)	Mass accuracy (△ppm)	
16	genkwanin-5-O-*β*-D-primeveroside	C_27_H_30_O_14_	4.00	[M+H]^+^	579.1706	579.1708	–0.35	431.1313, 285.0753, 271.0595
17	luteoloside	C_21_H_20_O_11_	4.03	[M+H]^+^	449.1074	449.1078	–0.89	287.0548, 271.0598, 153.0181, 127.0390
18	astragalin	C_21_H_20_O_11_	4.03	[M+H]^+^	449.1074	449.1078	–0.89	287.0548, 271.0598, 153.0181, 127.0390
19	3-O-*β*-D-glucoside-kaempferol	C_21_H_20_O_11_	4.03	[M+H]^+^	449.1074	449.1078	–0.89	287.0548, 271.0598, 153.0181, 127.0390
20	vitexin	C_21_H_20_O_10_	4.04	[M+H]^+^	433.1133	433.1129	0.92	415.1021, 397.0919, 367.0819, 337.0706, 313.0706, 283.0600, 271.0593
21	apigenin-7-O-*β*-D-glucose	C_21_H_20_O_10_	4.04	[M+H]^+^	433.1133	433.1129	0.92	415.1021, 397.0919, 367.0819, 351.0870, 313.0706, 297.0759, 283.0600, 271.0593
22	5-hydroxy-6,4′-dimethoxyflavone-7-O-*β*-D-glucoside	C_23_H_24_O_11_	4.21	[M+H]^+^	477.1388	477.1391	–0.63	315.0862, 300.0627, 272.0674
23	5,4′-dihydroxy-7,3′-dimethoxyluteolin	C_17_H_14_O_6_	4.22	[M+H]^+^	315.0858	315.0863	–1.59	300.0626, 272.0679, 257.0433, 167.0340
24	genkwanin-5-O-*β*-D-glucoside	C_22_H_22_O_10_	4.24	[M+H]^+^	447.1280	447.1285	–1.12	285.0757, 271.0596, 242.0571, 127.0387, 109.0280
No	Compounds	Formula	Retention time (min)	Identity	Precursor ion			Fragment ions (*m/z*)
					Experimental (*m/z*)	Theoretical (*m/z*)	Mass accuracy (△ppm)	
25	kaempferol-3-O-glucorhamnoside	C_27_H_30_O_15_	4.32	[M+H]^+^	595.1658	595.1657	0.17	301.0706, 271.0590, 4,109.0282
26	5, 6-dihydroxy flavanone - 7-o-glucoside acid	C_21_H_20_O_11_	4.42	[M+H]^+^	449.1088	449.1078	2.23	273.0758, 171.0285, 153.0183, 121.0646
27	isodaphnoretin	C_19_H_12_O_7_	4.51	[M+H]^+^	353.0654	353.0655	–0.28	192.0417, 177.0181, 163.0387, 161.0240, 146.0362, 135.0441, 133.0285
28	*α*-cyperone	C_15_H_22_O	4.57	[M+H]^+^	219.1743	219.1743	–0.18	175.1481, 161.1324, 133.1013, 119.0854, 107.0854, 105.0700
29	genistin	C_21_H_20_O_10_	4.59	[M+H]^+^	433.1133	433.1129	0.92	271.0601, 153.0181, 127.0389
30	cosnosiin	C_21_H_20_O_10_	4.59	[M+H]^+^	433.1133	433.1129	0.92	271.0601, 153.0181, 127.0389
31	apigenin	C_15_H_10_O_5_	4.62	[M+H]^+^	271.0600	271.0601	0.48	243.0653, 153.0183, 119.0492
32	genkwanin	C_16_H_12_O_5_	4.77	[M+H]^+^	285.0757	285.0758	0.35	270.0521, 257.0812, 242.0572, 197.0594, 167.0339, 124.0156
33	oroxylin	C_16_H_12_O_5_	4.77	[M+H]^+^	285.0757	285.0758	0.35	270.0521, 257.0812, 242.0572, 197.0594, 167.0339, 124.0156,
34	wogonin	C_16_H_12_O_5_	4.77	[M+H]^+^	285.0757	285.0758	0.35	270.0521, 257.0812, 242.0572, 197.0594, 167.0339, 124.0156
No	Compounds	Formula	Retention time (min)	Identity	Precursor ion	Fragment ions (*m/z*)		
					Experimental (*m/z*)	Theoretical (*m/z*)	Mass accuracy (△ppm)	
35	calycosin	C_16_H_12_O_5_	4.77	[M+H]^+^	285.0757	285.0758	0.35	270.0521, 257.0812, 242.0572, 225.0549
36	tiliroside	C_30_H_26_O_13_	4.78	[M+H]^+^	595.1437	595.1446	–1.51	309.0966, 287.0547, 165.0545, 147.0439, 119.0491,
37	kaempferol	C_15_H_10_O_6_	4.80	[M+H]^+^	287.0546	287.0550	–1.39	269.0438, 259.0596, 243.0636, 231.0650, 213.0544, 153.0182
38	luteolin	C_15_H_10_O_6_	4.80	[M+H]^+^	287.0546	287.0550	–1.39	269.0438, 259.0596, 241.0494, 213.0544, 153.0182, 135.0440, 127.0388
39	scutellarein	C_15_H_10_O_6_	4.80	[M+H]^+^	287.0546	287.0550	–1.39	269.0438, 259.0596, 241.0494, 171.0285, 153.0182, 125.0236
40	7-methoxy-luteolin-5-O-*β*-D-glucoside	C_22_H_22_O_11_	5.38	[M+H]^+^	463.1229	463.1234	–1.08	301.0705, 287.0544, 258.0527, 167.0335,
41	wogonoside	C_22_H_20_O_11_	5.48	[M+H]^+^	461.1084	461.1078	1.30	271.0601
42	melaleuca papyrin a-7-O-*β*-D-glucoside acid	C_22_H_20_O_11_	5.48	[M+H]^+^	461.1084	461.1078	1.30	271.0601
43	sakuranetin	C_16_H_14_O_5_	5.51	[M+H]^+^	287.0914	287.0914	0.00	137.0235
44	hydroxygenkwanin	C_16_H_12_O_6_	5.92	[M+H]^+^	301.0707	301.0707	0.07	286.0471, 258.0521, 241.0489, 167.0339, 135.0439, 124.0155
45	hispidulin	C_16_H_12_O_6_	5.92	[M+H]^+^	301.0700	301.0707	–2.33	286.0471, 258.0521, 241.0489
No	Compounds	Formula	Retention time (min)	Identity	Precursor ion			Fragment ions (*m/z*)
					Experimental (*m/z*)	Theoretical (*m/z*)	Mass accuracy (△ppm)	
46	3, 7-dimethoxy-5, 4′-dihydroxyflavone	C_17_H_14_O_6_	6.38	[M+H]^+^	315.0858	315.0863	–1.59	300.0626, 285.0375, 272.0676, 257.0435, 167.0336
47	3′, 4′-dihydroxy-3, 7-dimethoxyflavone	C_17_H_14_O_6_	6.38	[M+H]^+^	315.0858	315.0863	–1.59	300.0628, 272.0676, 285.0375, 257.0435
48	3′, 4′-dihydroxy-3, 8-dimethoxyflavone	C_17_H_14_O_6_	6.38	[M+H]^+^	315.0858	315.0863	–1.59	300.0628, 272.0676, 285.0375, 257.0435
49	5, 7-dihydroxy-6, 8-dimethoxyflavone	C_17_H_14_O_6_	6.38	[M+H]^+^	315.0858	315.0863	–1.59	300.0628, 272.0676, 285.0375, 257.0435
50	yuanhuafine	C_29_H_32_O_10_	7.82	[M+H]^+^	541.2070	541.2068	0.37	487.1727, 359.1476, 341.1390, 323.1280, 313.1433, 295.1329
51	yuanhuatine	C_34_H_34_O_10_	7.98	[M+H]^+^	603.2220	603.2224	–0.66	359.1485, 341.1387, 323.1276, 319.1168, 313.1436, 295.1331
52	genkwadaphnine	C_34_H_34_O_10_	7.98	[M+H]^+^	603.2220	603.2224	–0.66	359.1485, 341.1387, 323.1276, 319.1168, 313.1436, 295.1331
53	daphne diterpene ester-7	C_37_H_46_O_11_	9.51	[M+H]^+^	667.3117	667.3112	0.75	631.2869, 545.2733, 509.2534, 377.1599, 359.1483, 341.1381, 323.1279, 313.1436, 295.1327
54	yuanhuadine	C_32_H_42_O_10_	9.64	[M+H]^+^	587.2848	587.2850	–0.34	569.2775, 551.2692, 509.2544, 491.2425, 359.1486, 341.1381, 331.1556, 323.1274, 313.1424, 295.1327
No	Compounds	Formula	Retention time (min)	Identity	Precursor ion			Fragment ions (*m/z*)
					Experimental (*m/z*)	Theoretical (*m/z*)	Mass accuracy (△ppm)	
55	yuanhuacine	C_37_H_44_O_10_	10.64	[M+H]^+^	649.3001	649.3007	0.92	359.1476, 341.1381, 323.1273, 313.1431, 295.1325
56	genkwanine H	C_34_H_40_O_10_	11.41	[M+H]^+^	609.2666	609.2694	–4.60	591.2605
57	Yuanhuapine	C_29_H_34_O_10_	11.86	[M+H]^+^	543.2227	543.2225	0.37	507.2020, 471.1787, 447.1801, 361.1631
58	7-ketositosterol	C_29_H_48_O_2_	14.80	[M+H]^+^	429.3725	429.3727	–0.47	411.3622, 369.3159, 271.2055, 231.1741, 217.1573, 203.1431, 189.1277

#### Flavonoids

**FIGURE 4:**
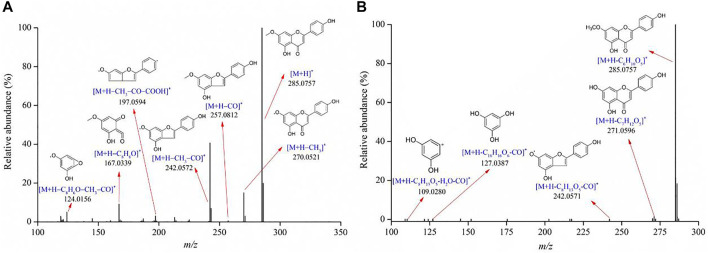
Product ion spectra of **(A)** genkwanin and **(B)** genkwanin-5-O-*β*-D-glucoside.

Flavonoids are one of the largest classes of small molecular bioactive compounds in plants. A total of 35 flavonoids were identified in the *Genkwa Flos*/glycyrrhizic acid self-assembled complex, including genkwanin, hydroxygenkwanin, genkwanin-5-O-*β*-D-primeveroside, genkwanin-5-O-*β*-D-glucoside, *etc*. (Table 1). The main mass spectrometric fragmentation mechanisms for flavonoids are neutral loss, retro-Diels-Alder (RDA) reaction, and glycosyl bond cleavage. The product ion spectrum of genkwanin shown in Figure 4 is representative of the flavonoid compounds. A protonated molecule [M+H]^+^ with a *m/z* of 285.0757 was detected in the positive ion mode. The protonated molecule lost a CO moiety from the C-ring, forming a fragment ion [M+H–CO]^+^ at *m/z* 257.0812. By further losses of CH_3_ and COOH, product ions [M+H–CO–CH_3_]^+^ at *m/z* 242.0572 and [M+H–CO–CH_3_–COOH]^+^ at *m/z* 197.0594 were observed. The fragment ion at *m/z* 270.0521 ([M+H–CH_3_]^+^) originated from the loss of CH_3_. Fragment ions at *m/z* 167.0339 were produced by RDA fragmentation in the C-ring of formononetin, which further eliminated the CH_3_ and CO, resulting in a fragment ion at *m/z* 124.0156. Another example is genkwanin-5-O-*β*-D-glucoside, which exhibited a protonated molecule [M+H]^+^ at *m/z* 447.1280 in the positive ion mode. By losing the glucose, CH_3_, CO, and H_2_O moieties or undergoing the RDA reaction, a series of product ions could be produced such as *m/z* 285.0757, 271.0596, 242.0571, 127.0387 and 109.0280. The proposed fragmentation patterns of diosmetin, apigenin, oroxylin, wogonin, calycosin, *etc* are shown in Supplementary Figure S3–S6.

#### Phenylpropanoids

**FIGURE 5:**
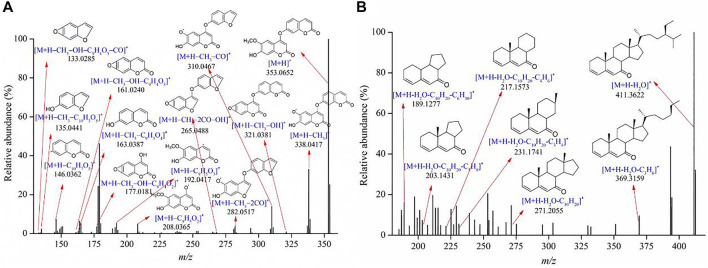
Product ion spectra of **(A)** isodaphnoretin and **(B)** 7-ketositosterol.

Some constituents belonging to the category of phenylpropanoids were also identified in the *Genkwa Flos*/glycyrrhizic acid self-assembled complex. The phenylpropanoids identified in this self-assembled complex included chlorogenic acids, neochlorogenic acid, cryptochlorogenic acid, 7-hydroxycoumarin, and isodaphnoretin. An investigation of their MS fragmentation patterns revealed common neutral losses of CO, CH_3_, and H_2_O. For example, isodaphnoretin exhibited a protonated molecule [M+H]^+^ at *m/z* 353.0652 in the positive ion mode. The protonated molecule lost a CH_3_ moiety, forming a fragment ion [M+H−CH_3_]^+^ at *m/z* 338.0417, which continued to lose a coumarin moiety to yield a fragment ion [M+H−CH_3_–C_9_H_3_O_4_]^+^ at *m/z* 163.0387. Due to consecutive losses of CO and OH, the ion at *m/z* 338.0417 produced more fragment ions at *m/z* 310.0467 ([M+H−CH_3_–CO]^+^), *m/z* 282.0517 ([M+H−CH_3_–2CO]^+^), and *m/z* 265.0488 ([M+H−CH_3_–2CO–OH]^+^). Likewise, the ion at *m/z* 321.0381 showed fragment ions at *m/z* of 177.0181, 161.0240, and 133.0285. Other fragments at *m/z* 208.0365 ([M+H−C_9_H_5_O_2_]^+^), 192.0417 ([M+H−C_9_H_5_O_3_]^+^), 146.0362 ([M+H−C_10_H_7_O_5_]^+^) and *m/z* 135.0441 ([M+H−CH_3_–C_10_H_3_O_5_]^+^) were also observed in the product ion spectrum. Figure 5A shows the proposed fragmentation pathway for isodaphnoretin. The proposed fragmentation patterns of chlorogenic acids, neochlorogenic acid, cryptochlorogenic acid, and 7-hydroxycoumarin are shown in Supplementary Figure S7.

#### Nucleosides

Some nucleosides were identified in the positive ion mode, including adenine, guanosine, iso-guanosine, and adenosine. Guanosine, which has a formula of C_10_H_13_N_5_O_5_, had a protonated molecule [M+H]^+^ at *m/z* 284.0987 (Fig. S8). The most intense product ion at *m/z* 152.0568 ([M+H–C_5_H_8_O_4_])^+^ was generated through the loss of a deoxyribofuranose, which was subjected to a further loss of NH_3_, producing a fragment ion at *m/z* 135.0302 ([M+H–C_5_H_8_O_4_–NH_3_]^+^). In addition, a fragment ion ([M+H–C_5_H_8_O_4_–CH_2_N_2_]^+^) at *m/z* 110.0350 was the product ion that resulted from the loss of imidazole from the ion *m/z* 152.0568. The proposed fragmentation patterns of adenine, guanosine, iso-guanosine, and adenosine are shown in Supplementary Figure S8.

#### Amino Acids

In this study, L-leucine and L-phenylalanine were also identified in the *Genkwa Flos*/glycyrrhizic acid self-assembled complex. L-leucine, which has an elemental composition of C_6_H_13_NO_2_, displayed a protonated molecule [M+H]^+^ at *m/z* 132.1018 in the positive ion mode (Supplementary Figure S8). The fragment ion at *m/z* 86.0965 was generated by decarboxylation, and further loss of NH_3_ produced a fragment ion of [M+H–HCOOH–NH_3_]^+^ at *m/z* 69.0700. Likewise, phenylalanine has the same cleavage pathway. The product ion spectrum of leucine and phenylalanine, along with possible identities of major ions, are displayed in Supplementary Figure S8

#### Terpenoids, Alkaloids, Aliphatic Aldehydes, and Essential Oils

Additional constituents were identified in the positive ion mode, including terpenoids, alkaloids, aliphatic aldehydes, and essential oils. As shown in Figure 5B, 7-ketositosterol formed the [M+H]^+^ ion at *m/z* 429.3725, which corresponds to the molecular formula C_29_H_48_O_2_. It yielded the fragment ion [M+H–H_2_O]^+^ at *m/z* 411.3622 [M+H–H_2_O–C_3_H_6_]^+^ at *m/z* 369.3159, and [M+H–H_2_O–C_10_H_20_]^+^ at *m/z* 271.2055 after the cracking of H_2_O, isobutane, and (R)-3-ethyl-2-methylheptane. Another characteristic fragment ion [M+H–H_2_O–C_10_H_20_–C_3_H_4_]^+^ at *m/z* 231.1741, was produced by the loss of one C_3_H_4_ molecule from *m/z* 271.2055. Due to consecutive losses of CH_2_, the ion at *m/z* 231.1741 produced more fragment ions at *m/z* 217.1573 ([M+H–H_2_O–C_10_H_20_–C_4_H_6_]^+^), *m/z* 203.1431 ([M+H–H_2_O–C_10_H_20_–C_5_H_8_]^+^), and *m/z* 189.1277 ([M+H–H_2_O–C_10_H_20_–C_6_H_10_]^+^). The proposed fragmentation pattern of trigonelline is shown in Supplementary Figure S8.

### Characterization of the Yuanhuacine/Glycyrrhizic Acid Self-Assembled Complex by ^1^H-Nuclear Magnetic Resonance and NOESY 2D Nuclear Magnetic Resonance

In an attempt to explore the possible formation of the *Genkwa Flos*/glycyrrhizic acid self-assembled complex, yuanhuacine, which is considered as both a toxic and an active compound [(Chen et al., 2012), (Chen et al., 2013)], and glycyrrhizic acid were further analyzed. Aqueous yuanhuacine and glycyrrhizic acid solutions were prepared at 80°C. The two aqueous solutions were then mixed at a 1:1 M ratio, resulting in the formation of a hydrogel complex. The same concentration of yuanhuacine and glycyrrhizic acid solution were prepared as a parallel control for subsequent analysis.

**FIGURE 6:**
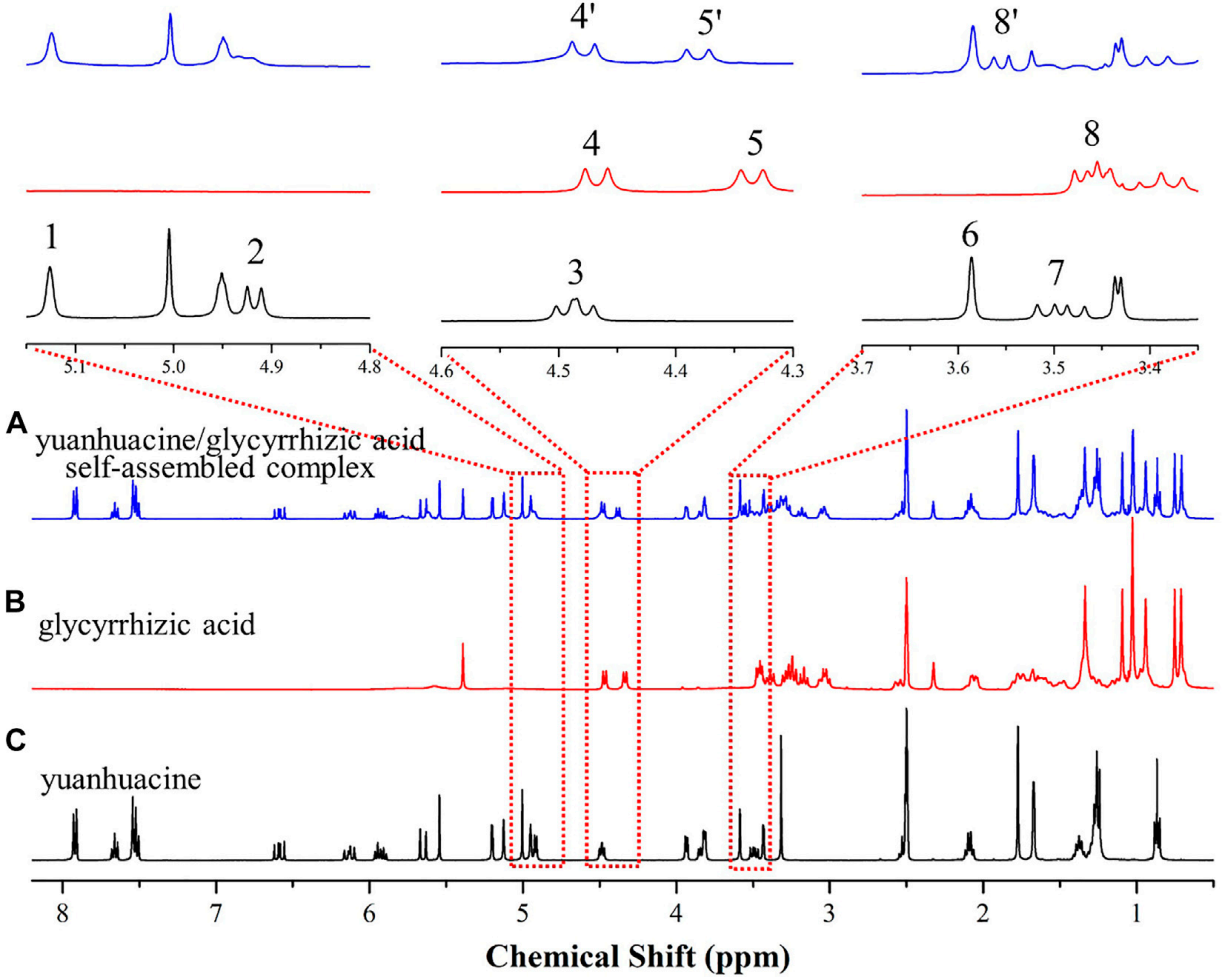
^1^H-NMR spectrum of **(A)** the yuanhuacine/glycyrrhizic acid self-assembled complex **(B)** glycyrrhizic acid, and **(C)** yuanhuacine.

The yuanhuacine, glycyrrhizic acid, and yuanhuacine/glycyrrhizic acid self-assembled complex were subjected to ^1^H-NMR characterization on a Bruker AVANCE III HD 400 MHz NMR spectrometer (Karlsruhe, Germany), as shown in Figure 6. It was found that after forming a self-assembled complex, the H-14 of yuanhuacine (Guo et al., 2014) disappeared in the low field, and the chemical shifts of GA’s Glu H-1′ (peak 4, from 4.48 to 4.49 ppm, Δ*δ* = −0.01 ppm) and Glu H-1″ (peak 5, from 4.34 to 4.39 ppm, Δ*δ* = −0.05 ppm) increased. Moreover, the chemical shifts of the H-20 in yuanhuacine moved low field from 3.50 (Yu et al., 2019) to 3.55 (8′) ppm and Glu-OH in GA moved low-field from 3.45 (Polyakov et al., 2008) to 3.55 (8′) ppm. Thus, it was initially speculated that the H-14 and H-20 of yuanhuacine and the Glu groups of GA were potential self-assembly binding sites. The results inferred the existence of the complex.

**FIGURE 7:**
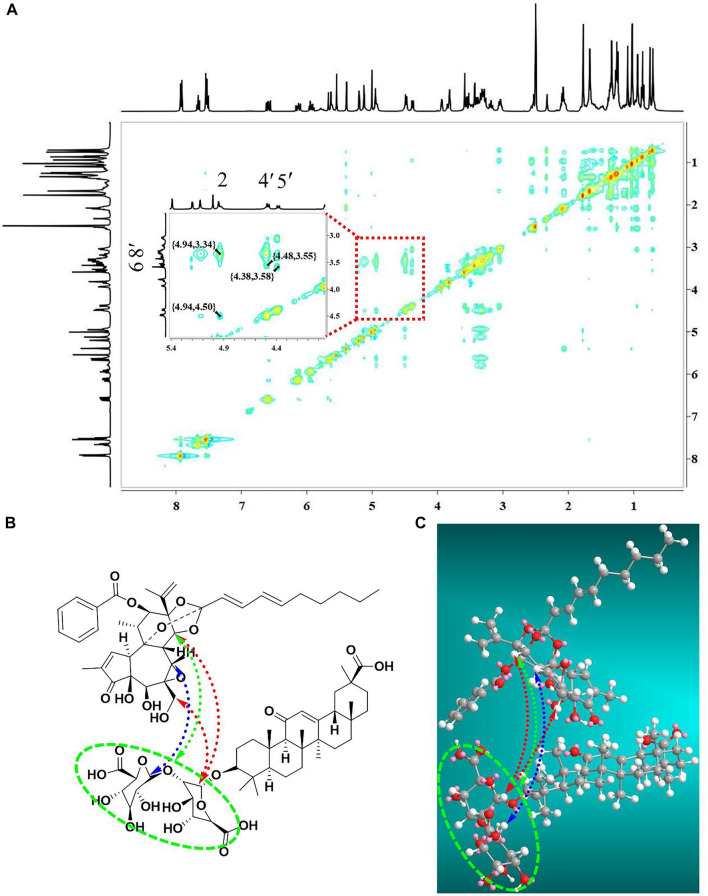
The self-assembly mechanism of the yuanhuacine/glycyrrhizic acid self-assembled complex **(A)** NOESY 2D ^1^H-NMR spectra of the yuanhuacine/glycyrrhizic acid self-assembled complex. **(B)** Chemdraw and **(C)** Chemdraw 3D of the yuanhuacine/glycyrrhizic acid self-assembled complex.

In addition, NOESY 2D NMR spectra were used to ensure the conformation of the complex units as well as the self-assembly mechanism (Figure 7A). In agreement with the ^1^H-NMR data, the NOESY spectrum showed strong interactions for the correlation signals {4.94, 3.34}, {4.48, 3.55}, {4.38, 3.58} and {4.94, 4.50}. Specifically, the hydrogen on H-14 (*δ* 4.94 ppm) of yuanhuacine correlated with the Glu-OH (*δ* 3.34 ppm) of GA; the hydrogens on H-20 (*δ* 3.55 ppm), and H-14 (*δ* 4.94 ppm) of yuanhuacine correlated with the Glu H-1′ (*δ* 4.48 ppm) of GA; and the hydrogen on Glu H-1″ (*δ* 4.38 ppm) of GA correlated with the H-7 (*δ* 3.58 ppm) of yuanhuacine. ChemBio 3D was used to predict the optimal conformation for the complex structure, as shown in Figure 7C. Based on the above evidence, we speculated that the mechanism was driven by hydrophobic interactions, which configured the hydrophobic pentacyclic triterpene of glycyrrhizic acid and yuanhuacine inward, and the hydrophilic glucuronic acid outward.

### Cytotoxicity Assay and Morphological Detection of Apoptosis Using DAPI Staining

**FIGURE 8:**
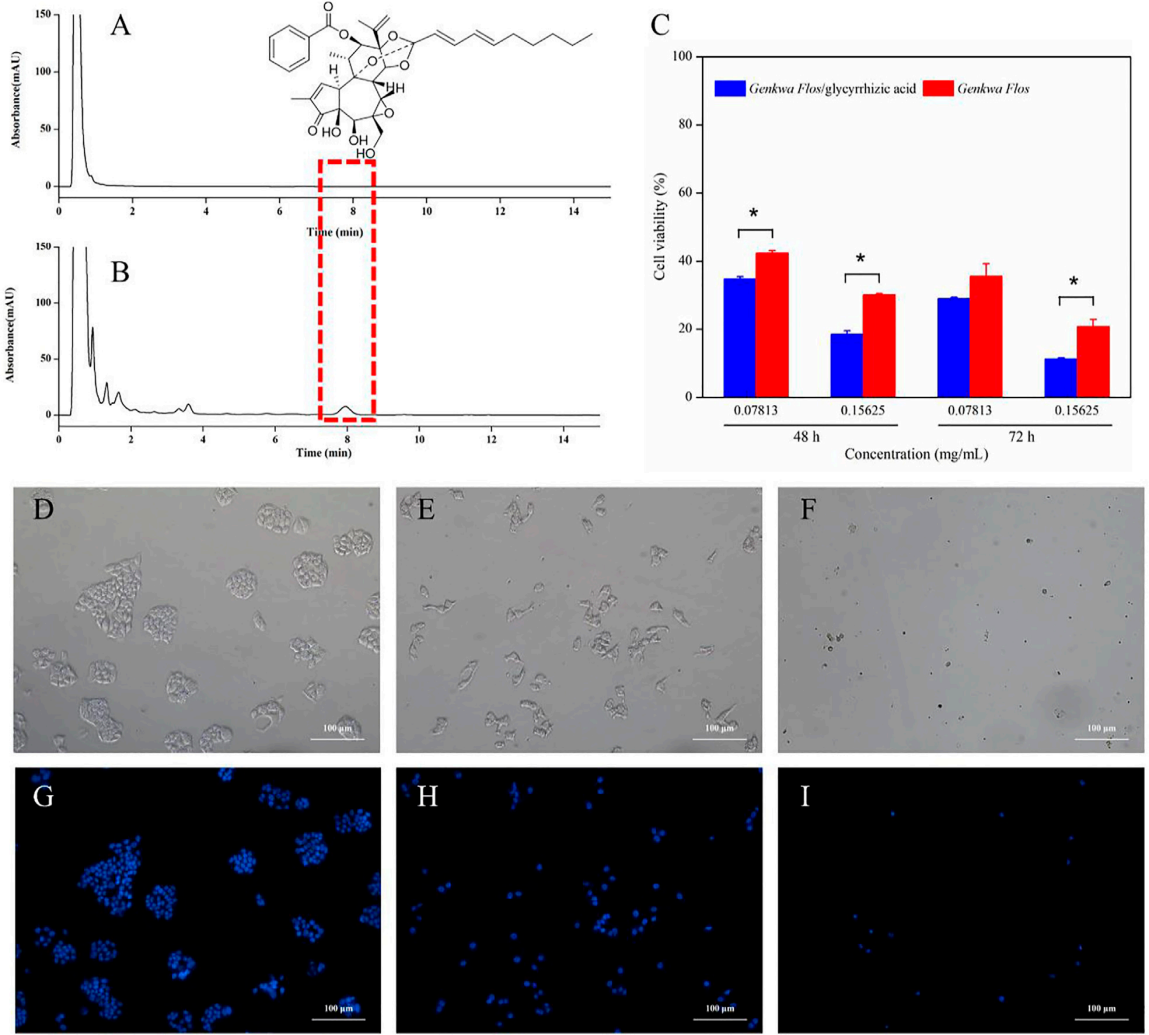
Chromatograms of **(A)** *Genkwa Flos* and **(B)** the *Genkwa Flos*/glycyrrhizic acid self-assembled complex **(C)** The *in vitro* cytotoxicity of *Genkwa Flos* and the *Genkwa Flos*/glycyrrhizic acid self-assembled complex against L-02 cells. *p < 0.05. **(D–I)** Morphological detection of apoptosis using DAPI staining (100 ×) on L-02 cells treated with *Genkwa Flos* and the *Genkwa Flos*/glycyrrhizic acid self-assembled complex: **(G)** control group, **(H)** 0.15625 mg/ml *Genkwa Flos*, and **(I)** 0.15625 mg/ml *Genkwa Flos*/glycyrrhizic acid self-assembled complex.

The *in vitro* cytotoxicities of *Genkwa Flos* and the *Genkwa Flos*/glycyrrhizic acid self-assembled complex were evaluated on L-02 cells using the MTT assay. The cell viability of *Genkwa Flos* and the *Genkwa Flos*/glycyrrhizic acid self-assembled complex are shown in Figure 8C. The *Genkwa Flos*/glycyrrhizic acid self-assembled complex was more cytotoxic against L-02 cells than *Genkwa Flos* with increasing concentration at 48 and 72 h, which was consistent with the increase in the dissolution of yuanhuacine, a toxic component, detected in HPLC (Figures 8A,B).

To further study their mechanism of growth inhibition, DAPI staining was performed after treating L-02 cells with 0.15625 mg/ml of *Genkwa Flos* and the *Genkwa Flos*/glycyrrhizic acid self-assembled complex for 72 h. As shown in Figures 8D,G, the L-02 control group exhibited intact cell bodies with clear, round nuclei that were stained blue. Meanwhile, the number of intact cells exposed to *Genkwa Flos* and the *Genkwa Flos*/glycyrrhizic acid self-assembled complex groups were significantly less, especially with those exposed to the *Genkwa Flos*/glycyrrhizic acid self-assembled complex group. The contours of some cells became irregular and the nuclear fluorescence was weak. Thus, the results indicated that *Genkwa Flos* and the *Genkwa Flos*/glycyrrhizic acid self-assembled complex could induce apoptosis in L-02 cells, but that the *Genkwa Flos*/glycyrrhizic acid self-assembled complex induced cell apoptosis more significantly.

## Conclusion

UHPLC-Q-Orbitrap HRMS and supramolecular self-assembly were utilized to reveal the toxicity-enhancing essence of glycyrrhizic acid on *Genkwa Flos*. The morphology and size distribution of the *Genkwa Flos*/glycyrrhizic acid self-assembled complex were characterized by SEM and DLS. In total, 58 compounds were identified within the supramolecular complex using accurate mass analysis, including eight diterpenoids, 35 flavonoids, five phenylpropanoids, four nucleosides, two amino acids, and four additional compounds. Their MS fragmentation pathways were further investigated. The formation mechanism of the yuanhuacine/glycyrrhizic acid self-assembled complex was fully investigated through ^1^H-NMR and NOESY 2D NMR analysis. This study provides fundamental scientific evidence of the presence of a self-assembled phytochemical supramolecular resulting from the combination of *Genkwa Flos* and glycyrrhizic acid, enabling better understanding of their clinical incompatibility and contraindication.

## Data Availability

The original data of UHPLC-Q-Orbitrap HRMS is available in the Mendeley database, DOI: 10.17632/4m866zfhfr.1
