# Supplementary material for: Revealing the Toxicity-Enhancing Essence of Glycyrrhiza on Genkwa Flos Based on Ultra-high-performance Liquid Chromatography Coupled With Quadrupole-Orbitrap High-Resolution Mass Spectrometry and Self-Assembled Supramolecular Technology
Source: Front Chem. 2021 Dec 23;9:740952. doi: 10.3389/fchem.2021.740952 (PMC8733466; doi:10.3389/fchem.2021.740952)
Supplement: Supplementary file 1 [file Presentation1.pdf]

Supporting Information

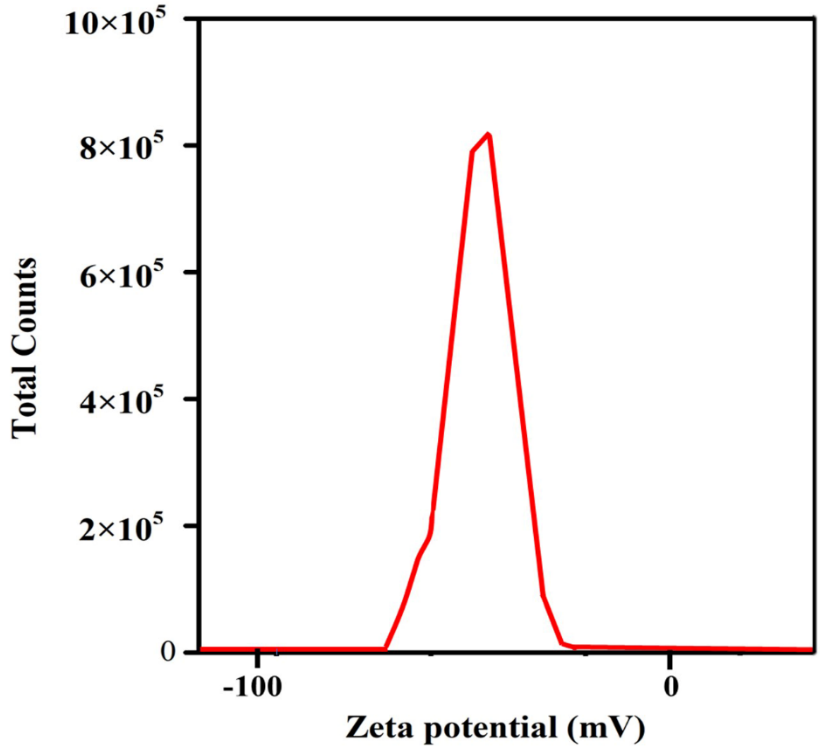

Fig. S1 Zeta potential of *Genkwa Flos*.

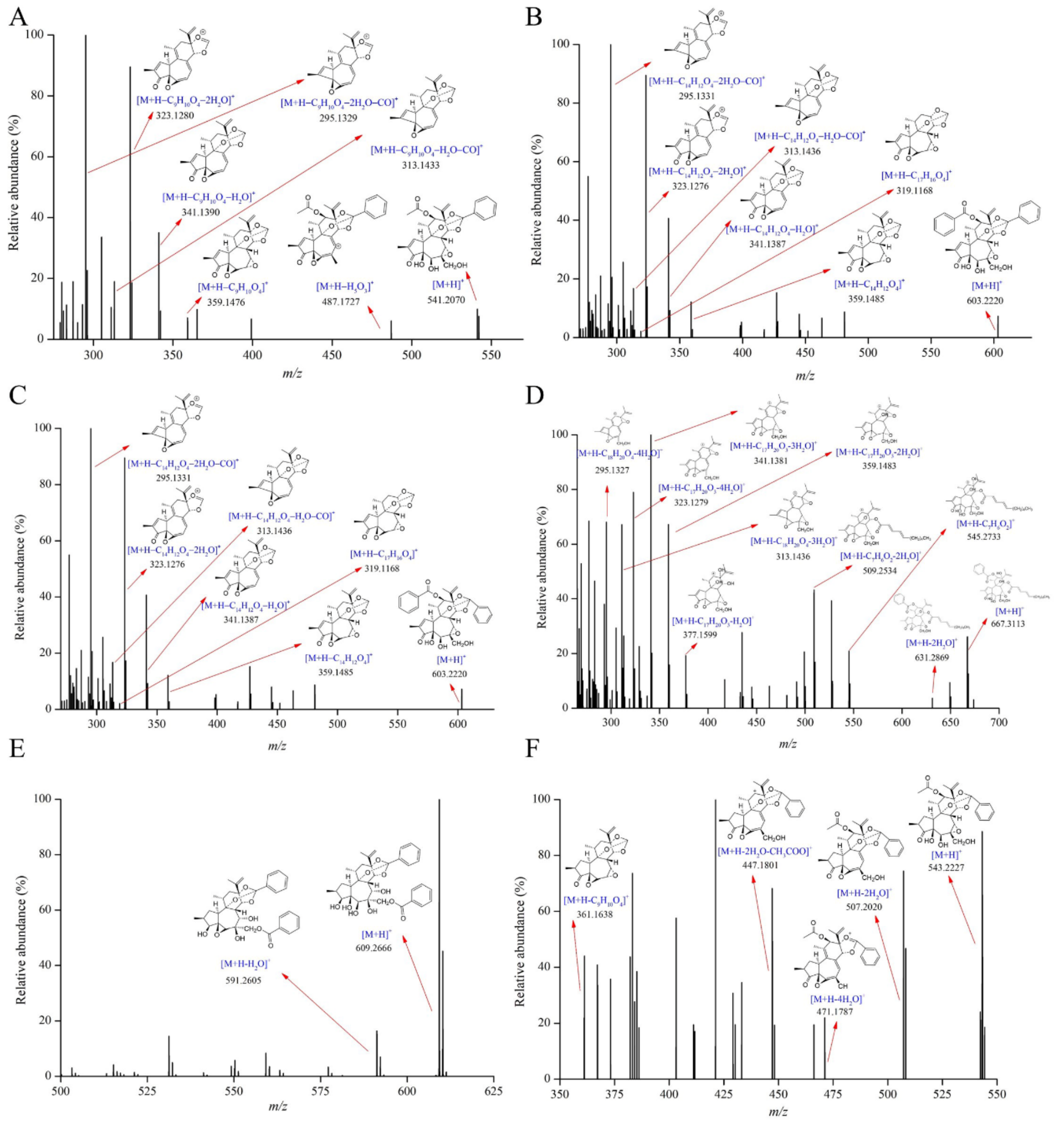

Fig. S2 Product ion spectra of A yuanhuafine; B yuanhuatine; C genkwadaphnin; D daphne diterpene ester-7; E genkwanine H; F yuanhuapine.

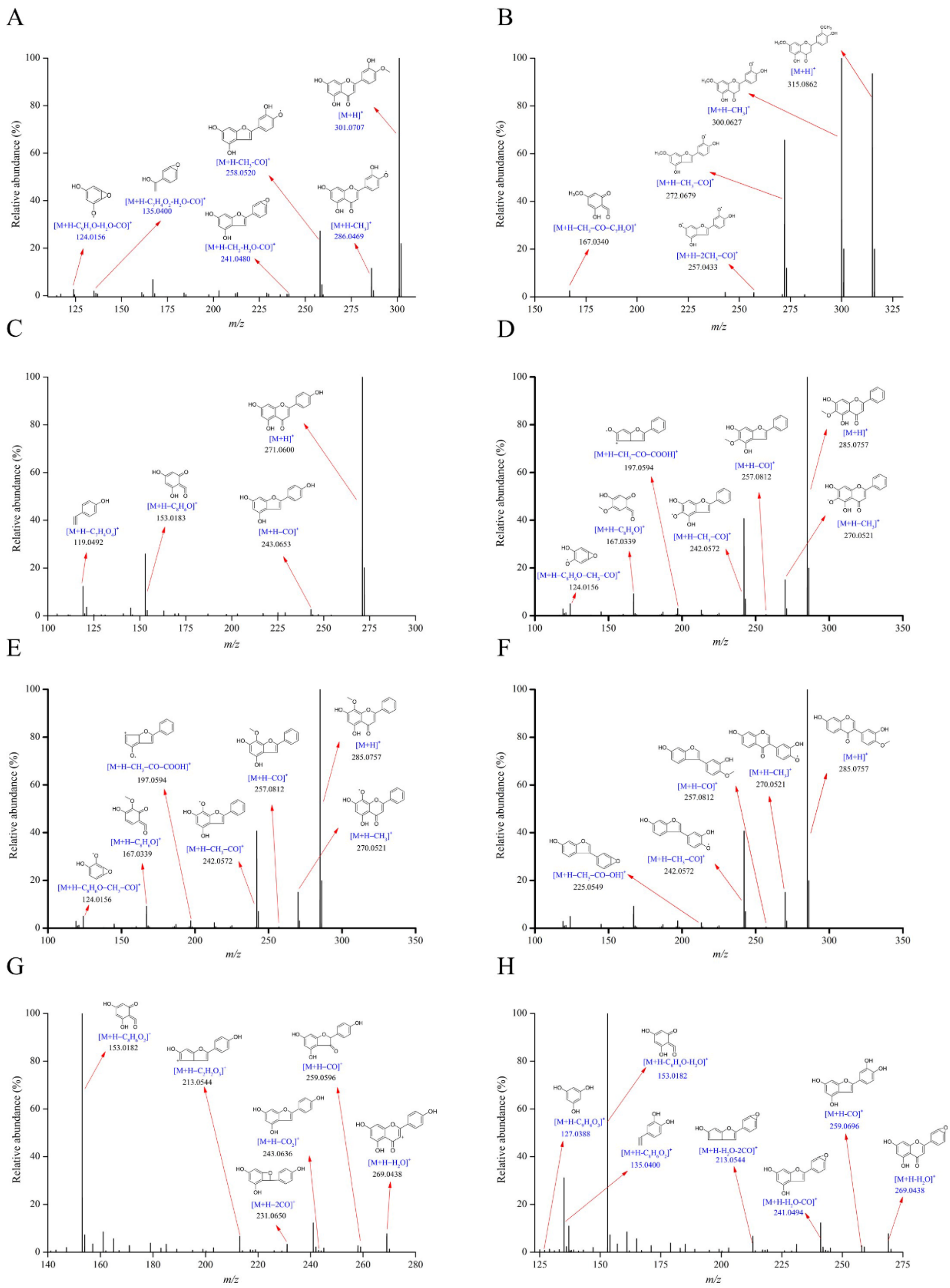

Fig. S3 Product ion spectra of A diosmetin; B 5,4'-dihydroxy-7,3'-dimethoxyluteolin; C apigenin; D oroxylin; E wogonin; F calycosin; G kaempferol; H luteolin.

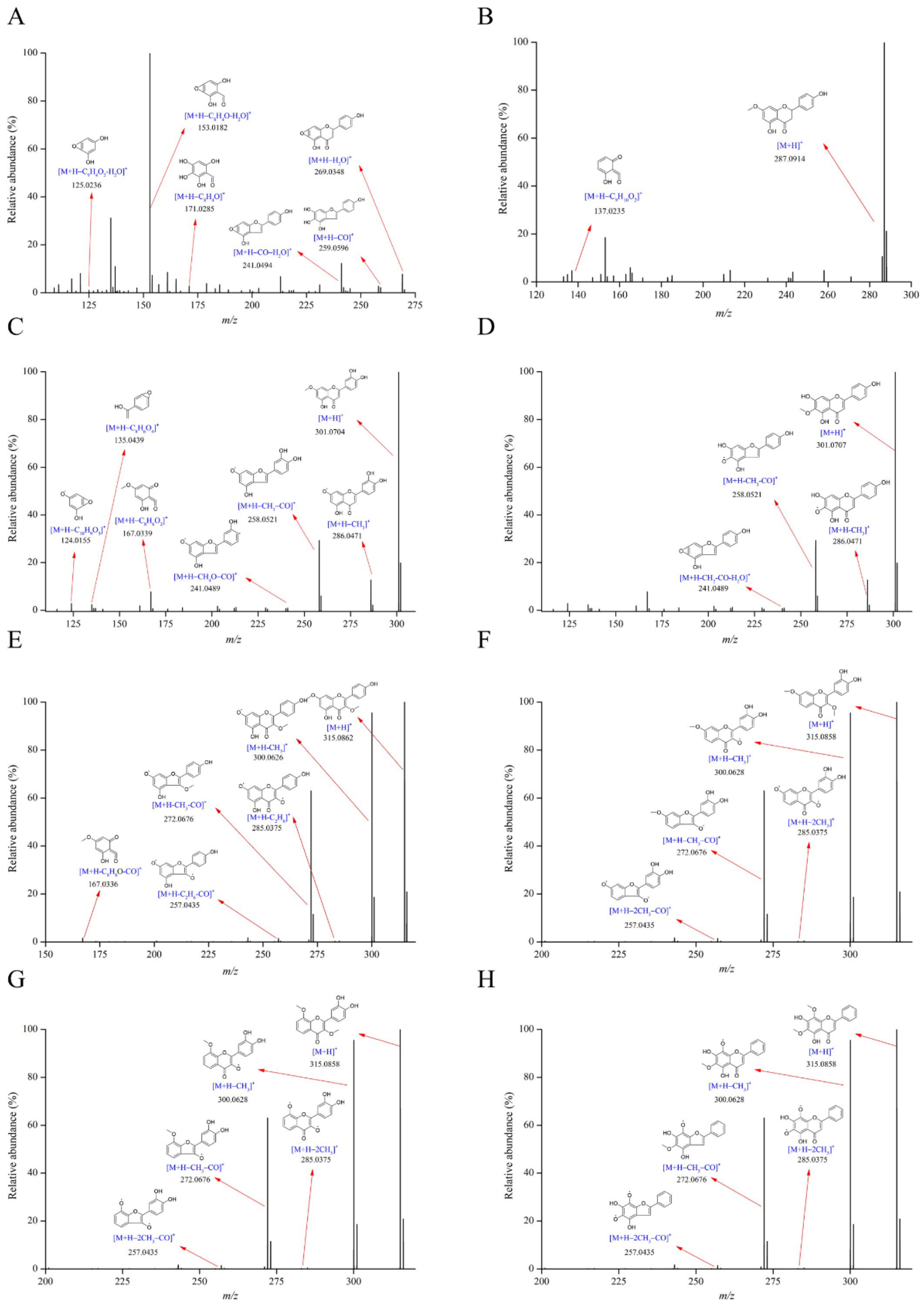

Fig. S4 Product ion spectra of A scutellarein; B sakuranetin; C hydroxygenkwanin; D hispidulin; E 3, 7-dimethoxy-5, 4'-dihydroxyflavone; F 3', 4'-dihydroxy-3, 7-dimethoxyflavone; G 3', 4'-dihydroxy-3, 8-dimethoxyflavone; H 5, 7-dihydroxy-6, 8-dimethoxyflavone.

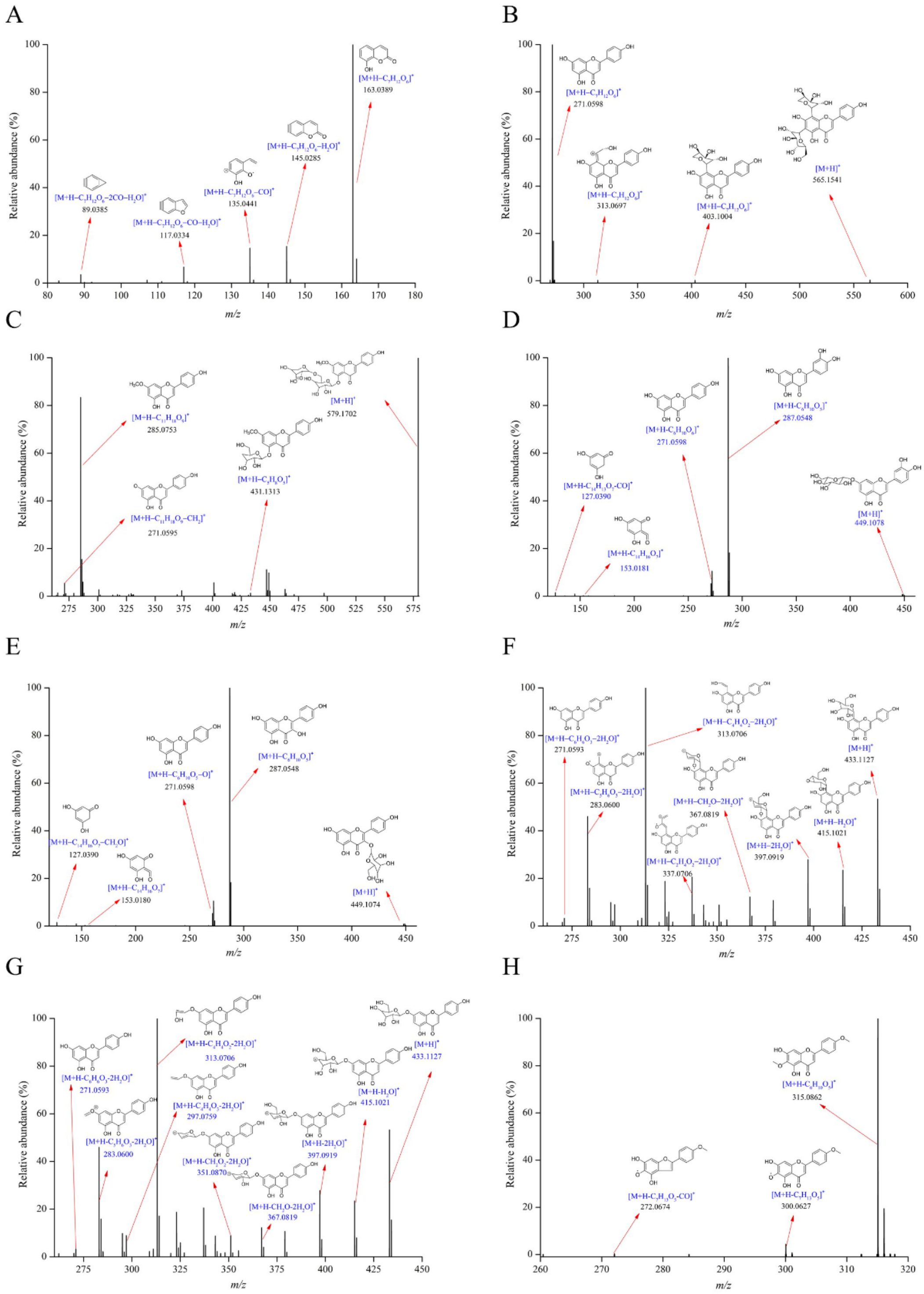

Fig. S5 Product ion spectra of A scopolin; B schaftoside; C daphne genkwanin-5-O-β-D-rubianprimrose glycoside; D luteoloside; E 3-O-β-D-glucoside - kaempferol; F vitexin; G apigenin -7-O-β-D-glucose; H 5-hydroxy-6,4'-dimethoxyflavone-7-O-β-D-glucoside.

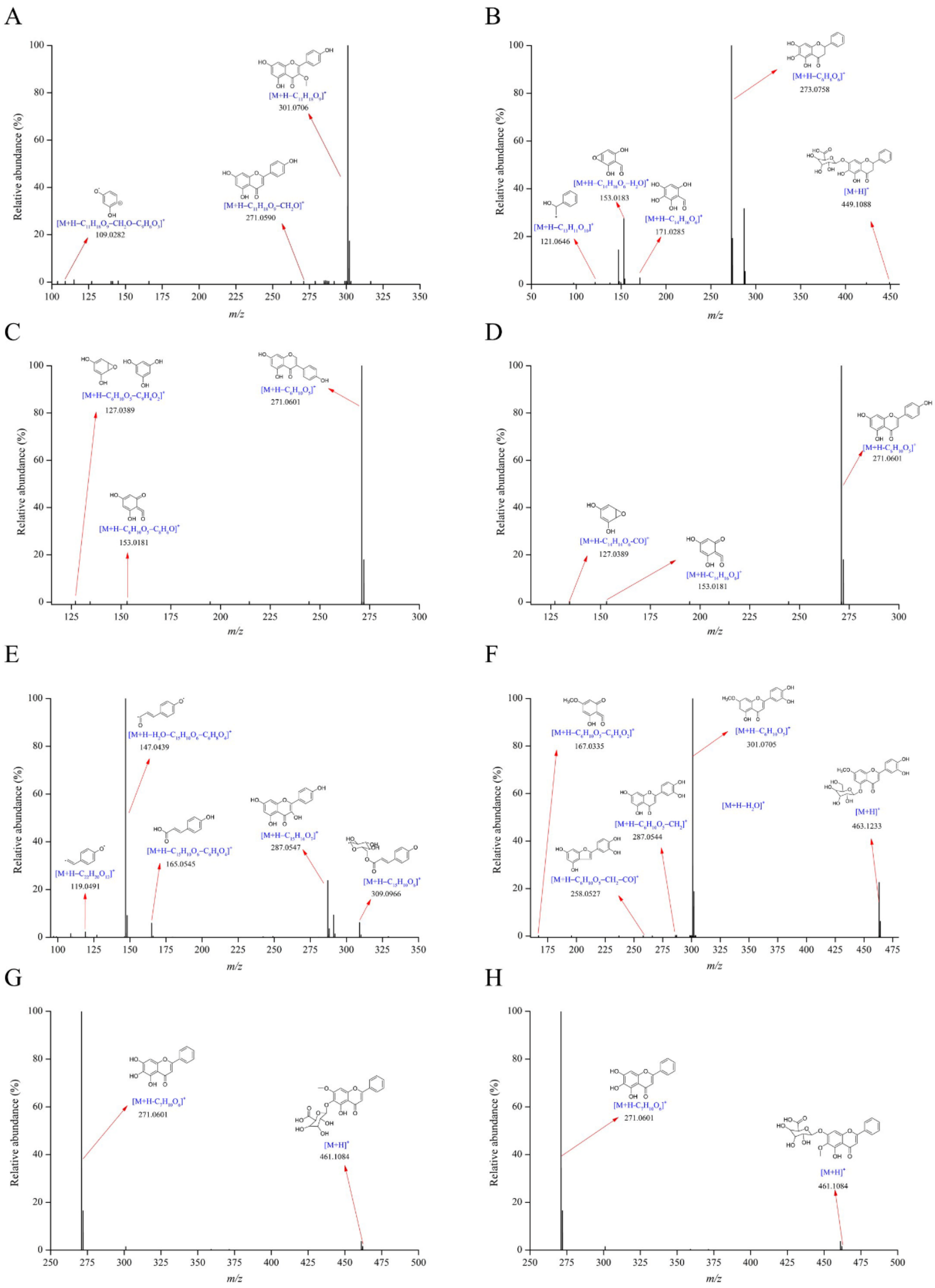

Fig. S6 Product ion spectra of A kaempferol-3-O-glucorhamnoside; B 5, 6-dihydroxy flavanone -7-O-glucoside acid; C genistin; D cosnosiin; E tiliroside; F 7-methoxy-luteolin-5-O-*β*-D-glucoside; G wogonoside; H melaleuca papyrina-7-O-*β*-D-glucoside acid.

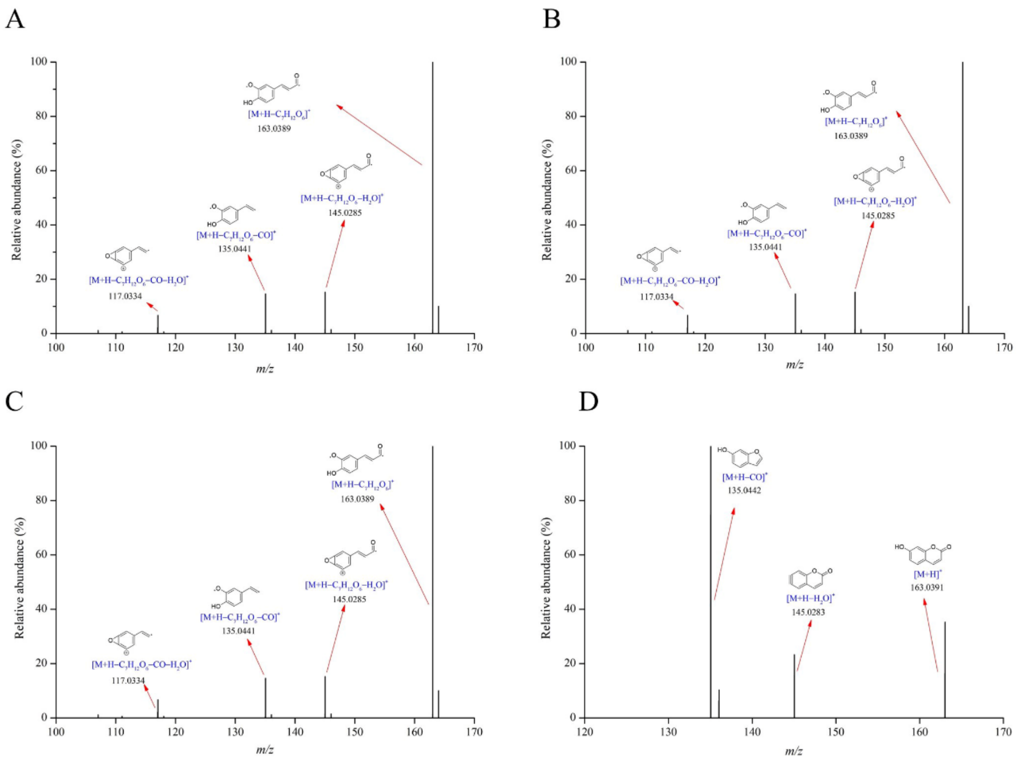

Fig. S7 Product ion spectra of A chlorogenic acids; B neochlorogenic acid; C cryptochlorogenic acid; D 7-hydroxycoumarin.

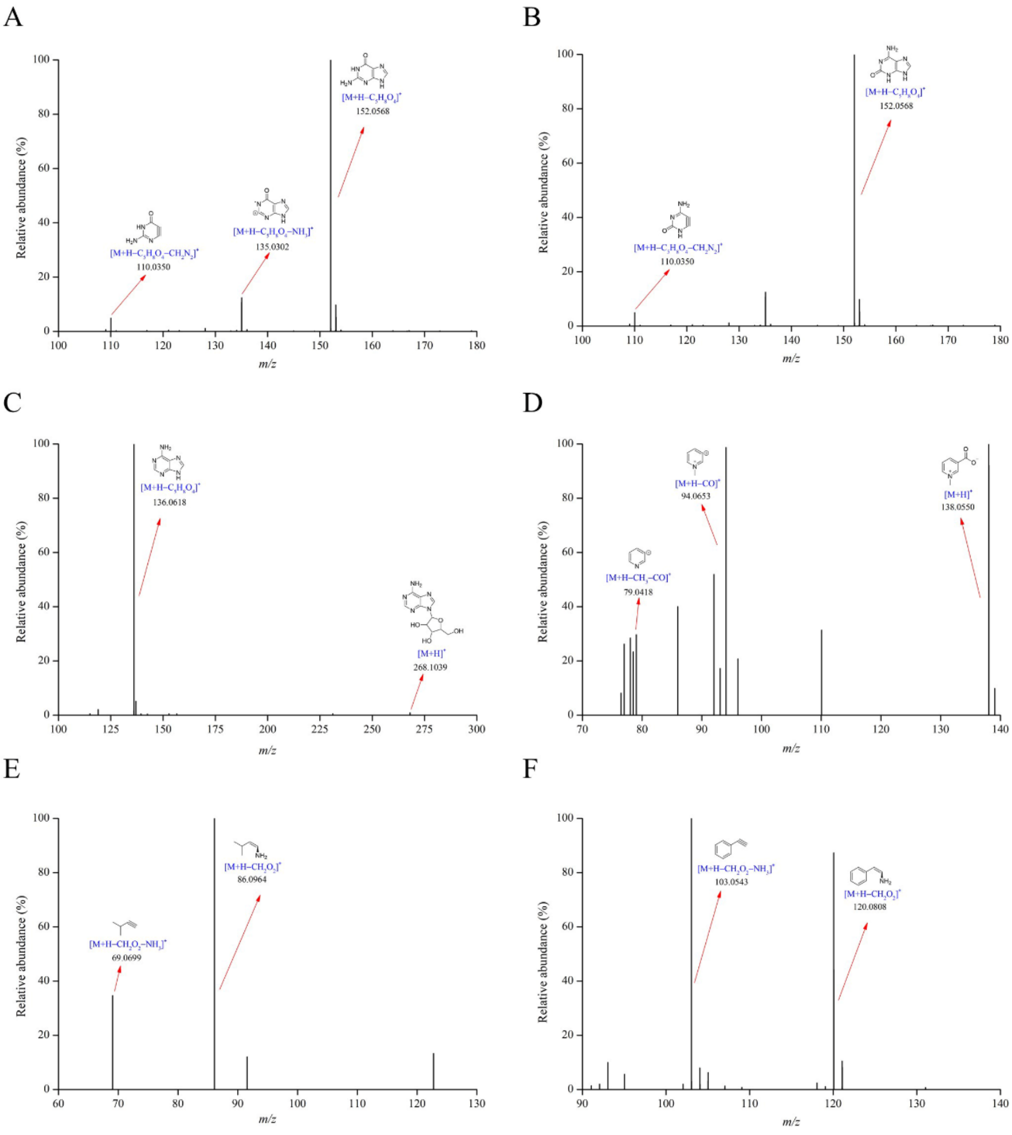

Fig. S8 Product ion spectra of A guanosine; B iso-guanosine; C adenosine; D trigonelline; E L-leucine; F L-phenylalanine.
